# Socioeconomic Determinants of Diabetes Self-Care and Health Literacy Among Adults in Southern Riyadh

**DOI:** 10.3390/healthcare14172724

**Published:** 2026-08-26

**Authors:** Mohammed Almutairi, Waleed M. Alshehri, Abdulaziz M. Alodhailah, Bader M. Almutairy

**Affiliations:** 1 Department of Medical Surgical Nursing, College of Nursing, King Saud University, Riyadh 11451, Saudi Arabia; 2 Community and Psychiatric Mental Health Nursing, College of Nursing, King Saud University, Riyadh 11451, Saudi Arabia

**Keywords:** social determinants of health, diabetes self-care, income inequality, aging, education, Saudi Arabia, health equity, primary healthcare

## Abstract

**Background/Objectives:** Social determinants of health, including income, age, education, and gender, have been proposed to shape diabetes self-care capacity, yet their joint associations within Saudi Arabian primary healthcare populations remain poorly delineated. Anchored within the World Health Organization’s Commission on Social Determinants of Health framework and the Social Ecological Model, this study examined both the unadjusted and, via multivariable regression, adjusted associations of four sociodemographic factors with diabetes self-care management and health literacy among adults in southern Riyadh. **Methods:** A cross-sectional correlational design was employed with 92 adults with type 2 diabetes recruited from primary healthcare centers in southern Riyadh. Data were collected using the Arabic HLS-Q12 (health literacy) and SDSCA-Arabic (self-care management). Non-parametric Spearman rank-order correlations were conducted to examine bivariate associations. Multivariable linear regression was subsequently performed to estimate adjusted associations for each sociodemographic variable. Bivariate analyses were conducted using IBM SPSS Statistics for Windows, Version 25.0 (IBM Corp., Armonk, NY, USA), while multivariable linear regression analyses were performed using Python version 3.12 with the statsmodels package. **Results:** Income level showed significant, moderate positive unadjusted correlations with both self-care management (rs = 0.40, 95% CI [0.21, 0.56], *p* < 0.01) and health literacy (rs = 0.41, 95% CI [0.22, 0.57], *p* < 0.01). Age showed the strongest negative unadjusted correlation with self-care management (rs = −0.49, 95% CI [−0.63, −0.31], *p* < 0.01) and a significant negative correlation with health literacy (rs = −0.36, 95% CI [−0.53, −0.17], *p* < 0.01). Educational attainment was significantly associated with both self-care management (rs = 0.29, 95% CI [0.09, 0.47], *p* < 0.01) and health literacy (rs = 0.26, 95% CI [0.06, 0.44], *p* < 0.05). Gender did not reach statistical significance for either outcome. A multivariable model adjusting for all four sociodemographic variables confirmed that income and age remained independently associated with both outcomes, whereas education and gender did not. These findings do not establish independent or causal effects from the bivariate correlations alone; the adjusted results are reported separately below. **Conclusions:** Income and age were independently associated with both diabetes self-care and health literacy after multivariable adjustment, whereas education and gender were not. Findings support equity-focused nursing interventions and age-responsive diabetes education attentive to the social patterning of self-care disparities in southern Riyadh.

## 1. Introduction

The social determinants of health, which are the conditions in which people are born, grow, live, work, and age, are widely acknowledged to exert a decisive influence over population health outcomes [1,2]. These factors frequently surpass the effects of clinical care in shaping trajectories of disease and well-being [2,3]. The WHO Commission on Social Determinants of Health (CSDH) framework, reaffirmed in the 2021 global report, situates socioeconomic position as the most consequential upstream driver of health inequities globally [2]. In the domain of chronic disease management, and specifically within the context of diabetes mellitus, the role of social determinants in shaping an individual’s capacity for effective self-care has attracted increasing scientific attention [4,5,6]. Self-care management in diabetes is a complex, sustained behavioral enterprise that requires individuals to engage in blood glucose monitoring, dietary modification, physical activity, foot care, and medication adherence on a daily basis. The execution of these activities is profoundly shaped by the economic, educational, and social resources available to the individual [7].

Saudi Arabia presents a compelling case study for examining these determinants. The Kingdom’s rapid economic development over the past four decades has generated substantial internal inequality, with significant disparities in income, educational attainment, and access to healthcare services persisting across geographic regions [8]. While universal health coverage for Saudi nationals reduces direct financial barriers, indirect costs such as monitoring supplies, specialized dietary foods, and loss of income during illness continue to stratify self-care quality [9,10,11]. Income exerts influence through multiple pathways, both directly through access to resources and indirectly through its effects on health literacy, stress levels, and quality of social support networks [12,13,14]. Studies in high-income countries and the Arabian Gulf have consistently documented income gradients in diabetes outcomes, where lower monthly household income is significantly associated with poorer glycemic control and reduced engagement in dietary self-care [15,16].

Health literacy occupies a central position within this social determinants framework. It is defined as the cognitive and social skills that determine the motivation and ability of individuals to gain access to, understand, and use information in ways that promote and maintain good health [2]. Functionally adequate health literacy enables patients to navigate complex healthcare systems and make autonomous decisions aligned with evidence-based recommendations [17]. Conversely, limited health literacy is independently associated with misinterpretation of medical instructions, suboptimal medication adherence, and elevated rates of preventable hospitalizations [18,19,20]. In Saudi Arabia, studies have reported variable prevalence rates of limited health literacy ranging from 29% to 52%, often patterned by sociodemographic characteristics [21].

Age, educational attainment, and gender constitute distinct social determinants that intersect with these literacy levels. Older adults face a constellation of challenges including age-related cognitive decline, sensory impairments, and limited digital literacy, which may compound difficulties in applying health information [22,23]. Educational attainment is also a critical factor, as the expansion of public education in Saudi Arabia since the 1970s has produced substantial heterogeneity across age cohorts [24]. However, education and health literacy are not interchangeable; educational attainment may independently influence self-care through occupational status and stronger health-promoting social networks [17]. Furthermore, gender roles and norms in Arab populations may influence self-care through differential access to health information and varying cultural expectations regarding physical activity and dietary practices [15].

Theoretically, this study is grounded in Orem’s Self-Care Deficit Nursing Theory (SCDNT), which posits that effective self-care is contingent upon individuals possessing sufficient self-care agency [25]. Health literacy is conceptualized as a constitutive dimension of this agency. When self-care agency is insufficient relative to the therapeutic self-care demand, a self-care deficit emerges, necessitating nursing intervention. This is further situated within the Social Ecological Model, which views individual behaviors within nested layers of interpersonal, community, and policy influence [26]. Together, these frameworks support a conceptualization of diabetes self-care disparities as the product of intersecting social forces rather than purely individual deficits.

Despite these established associations, critical gaps persist in the literature pertaining to Arabic-speaking populations in primary care. Southern Riyadh, characterized by lower average household incomes and a higher concentration of older adults with limited formal education, represents a population of distinct epidemiological relevance [27]. Yet, to our knowledge, no prior study has specifically examined the interplay between health literacy and diabetes self-care within this subpopulation using a comprehensive social determinants lens. This analytical gap constrains the ability of healthcare policymakers and nursing practitioners to design appropriately targeted interventions.

The present study therefore aimed to systematically examine the bivariate and, through multivariable regression, adjusted associations of income level, age, gender, and educational attainment with diabetes self-care management and health literacy among adults in southern Riyadh. By adopting an explicit social determinants lens, the study aspires to move beyond descriptive epidemiology toward actionable insights that can inform equity-focused nursing practice, health education, and policy in a rapidly urbanizing Saudi community.

## 2. Materials and Methods

### 2.1. Study Design and Theoretical Grounding

A cross-sectional, correlational quantitative design was employed. The study was conceptually anchored within the WHO CSDH framework and the Social Ecological Model, both of which frame individual health behaviors as the product of structural and contextual social forces. Within this theoretical architecture, income, age, gender, and educational attainment were treated as upstream structural factors hypothesized to be associated with both health literacy (an intermediary factor) and diabetes self-care management (the proximal behavioral outcome). Orem’s Self-Care Deficit Nursing Theory, introduced below, complements this structural framing by specifying the individual-level mechanism, self-care agency, through which these upstream social factors are theorized to relate to self-care behavior.

### 2.2. Setting and Sample

The study was conducted across primary healthcare centers (PHCs) in southern Riyadh, Saudi Arabia, a geographic area characterized by lower average income and educational attainment relative to other metropolitan districts. Required sample size was determined a priori using G*Power 3.1 for a multiple linear regression with five predictors (α = 0.05, power = 0.80, f^2^ = 0.15, medium effect size), yielding a minimum required sample of 92 participants; the multivariable model ultimately reported in Section 3.5 includes four of these predictors (income, age, education, and gender), as no fifth conceptually justified sociodemographic predictor was retained for the final model. A total of 120 adults with confirmed type 2 diabetes diagnoses were recruited via purposive sampling; 28 surveys were subsequently excluded because missing responses exceeded 30% of total questionnaire items, yielding a final analytic sample of 92 participants. Inclusion criteria required participants to be aged 18 years or older, fluent in Arabic, attending a southern Riyadh PHC during the data collection period, and free of psychiatric diagnoses. Data collection was facilitated through SurveyMonkey™ (SurveyMonkey Inc., San Mateo, CA, USA) to ensure participant anonymity.

### 2.3. Measures

Diabetes self-care management was assessed using the SDSCA-Arabic (8 items; Al Shehri et al., [28]), which captures the frequency of self-care behaviors over the preceding seven days across dietary, physical activity, blood glucose monitoring, and foot care domains (Cronbach’s α = 0.81). Health literacy was measured using the HLS-Q12-Arabic (12 items; Awwad et al., [29]), yielding scores from 12 to 48 across four competency domains (Cronbach’s α = 0.850). Sociodemographic data including age, gender, educational level, monthly income, marital status, employment status, and diabetes disease duration were collected via a structured demographic questionnaire.

### 2.4. Statistical Analysis

Distributional normality was assessed via the one-sample Kolmogorov–Smirnov test with Lilliefors correction and confirmed with the Shapiro–Wilk test, both indicating non-normal distributions for self-care management (Lilliefors *p* = 0.005; Shapiro–Wilk *p* = 0.003) and health literacy (Lilliefors *p* = 0.007; Shapiro–Wilk *p* = 0.025). Spearman rank-order correlations (rs) were computed to examine bivariate associations between each sociodemographic predictor and the outcome variables (self-care management and health literacy), with the results presented in Table 1. To estimate adjusted, independent associations, multivariable linear regression models were subsequently fitted for each outcome, with income, age, education level, and gender entered simultaneously as predictors; model diagnostics (variance inflation factors, residual normality, and homoscedasticity) were examined, and bias-corrected bootstrap confidence intervals (5000 resamples) were computed as a robustness check where residual normality was not fully met. For inferential purposes, income and age were entered into the Spearman correlation analyses as their original continuous values. The categorical groupings used for descriptive subgroup comparison in Table 2 and Table 3 (income: low, <5000 SAR/month; middle, 5000–9999 SAR/month; higher, ≥10,000 SAR/month; age: 18–35, 36–50, 51–65, >65 years) were not themselves entered into the correlation analyses. Because gender was recorded as a binary variable, the Spearman correlation coefficient reported for gender is mathematically equivalent to a rank-biserial correlation between the dichotomous grouping and the continuous outcome. Given that eight bivariate correlations were examined across two outcomes, no correction for multiple comparisons was applied; results should therefore be interpreted with awareness of the increased risk of Type I error, consistent with an exploratory rather than confirmatory analytic framework. All analyses were conducted with SPSS Version 25.0 at α = 0.05.

## 3. Results

### 3.1. Sociodemographic Profile

The sample included 92 adults with diabetes (54.35% female, 45.65% male; mean age = 42.95 years, SD = 14.61). The majority were married (73.91%), and the most prevalent educational category was secondary education (34.78%), followed by higher education (32.61%). Less than 37% were employed at the time of data collection. Mean diabetes disease duration was 12.10 years (SD = 8.73), and mean monthly income was 7270.23 SAR (SD = 5621.90).

### 3.2. Income and Self-Care and Health Literacy Outcomes

Income showed significant, moderate positive correlations with both diabetes self-care management (rs = 0.40, 95% CI [0.21, 0.56], *p* < 0.01) and health literacy (rs = 0.41, 95% CI [0.22, 0.57], *p* < 0.01). Subgroup means, presented in Table 2, showed a progressive increase across income categories for both outcomes; a Kruskal–Wallis test confirmed significant between-groups differences across the three income categories for both self-care management (H(2) = 13.45, *p* = 0.001) and health literacy (H(2) = 14.97, *p* < 0.001), supporting a descriptive social-gradient interpretation, although this omnibus test does not itself establish a linear trend or adjust for other variables.

### 3.3. Age and Self-Care and Health Literacy Outcomes

Age showed the strongest negative correlation with diabetes self-care management of all sociodemographic variables examined (rs = −0.49, 95% CI [−0.63, −0.31], *p* < 0.01), a moderate-to-strong association by conventional interpretive benchmarks for correlation coefficients. Age also showed a significant, moderate negative correlation with health literacy (rs = −0.36, 95% CI [−0.53, −0.17], *p* < 0.01). Descriptive subgroup means, presented in Table 3, showed a consistent decline in both self-care and health literacy scores across successively older age categories. This pattern is descriptively substantial; however, because no formal trend test, adjusted model, or clinically validated minimally important difference was available for these outcomes, we describe it as a marked descriptive gradient rather than as evidence of clinical significance.

### 3.4. Education, Gender, and Outcomes

Educational attainment showed significant, weak-to-moderate positive correlations with both self-care management (rs = 0.29, 95% CI [0.09, 0.47], *p* < 0.01) and health literacy (rs = 0.26, 95% CI [0.06, 0.44], *p* < 0.05); possible explanatory pathways are proposed as hypotheses in the Discussion. Gender was not significantly correlated with either self-care management (rs = 0.11, 95% CI [−0.10, 0.31], *p* > 0.05) or health literacy (rs = −0.02, 95% CI [−0.22, 0.19], *p* > 0.05); this pattern is considered further in Section 4.4.

### 3.5. Multivariable Analysis of Sociodemographic Associations

To estimate adjusted, independent associations, multivariable linear regression models were fitted for self-care management and health literacy, with income, age, education level, and gender entered simultaneously (Table 4). For self-care management, the overall model was significant, F(4,87) = 12.60, *p* < 0.001, R^2^ = 0.367, adjusted R^2^ = 0.338. Income (B = 8.5 × 10^−5^, β = 0.295, *p* = 0.002) and age (B = −0.045, β = −0.401, *p* < 0.001) remained independently associated with self-care management after adjustment; education showed a marginal, attenuated association (β = 0.175, *p* = 0.056), and gender remained non-significant (β = 0.107, *p* = 0.221).

For health literacy, the overall model was also significant, F(4,87) = 7.49, *p* < 0.001, R^2^ = 0.256, adjusted R^2^ = 0.222. As with self-care management, income (B = 0.0004, β = 0.350, *p* = 0.001) and age (B = −0.138, β = −0.306, *p* = 0.002) remained independently associated with health literacy after adjustment, while education (β = 0.037, *p* = 0.707) and gender (β = −0.046, *p* = 0.629) did not. Because the health-literacy model’s residuals departed from normality (Shapiro–Wilk *p* < 0.001), bias-corrected bootstrap confidence intervals (5000 resamples) were computed as a robustness check; these confirmed the asymptotic conclusions for every predictor, including the attenuation of education to non-significance. Variance inflation factors were below 1.2 for all predictors in both models, indicating no meaningful multicollinearity, and the self-care model’s residuals did not depart from normality (Shapiro–Wilk *p* = 0.120) or homoscedasticity (Breusch–Pagan *p* = 0.167).

Taken together, the adjusted models indicate that the bivariate associations of income and age with both outcomes are not attributable to overlap with education or gender: both remained significant predictors after mutual adjustment. In contrast, the bivariate association of education with both outcomes attenuated substantially after adjustment for income and age, suggesting that education’s unadjusted association may be partly explained by its overlap with income and age rather than reflecting an independent effect. Gender was not significantly associated with either outcome in either the bivariate or the adjusted analysis.

## 4. Discussion

### 4.1. The Income Gradient in Diabetes Self-Care

The identification of a significant, moderate positive correlation between income and both diabetes self-care management and health literacy is consistent with the theoretical propositions of the WHO CSDH framework [2]. The progressive pattern of subgroup means observed across income categories, wherein self-care and health literacy scores increased from low- to high-income groups, is descriptively consistent with a social-gradient interpretation, although this cross-sectional, unadjusted analysis cannot establish that material advantage causally confers self-care benefits. Unlike education, income’s association with both outcomes remained independently significant after adjustment for age, education, and gender in the multivariable model (Section 3.5), indicating that it is not simply a proxy for the other sociodemographic variables measured here. This pattern is broadly consistent with findings from Walker et al. [16] in North American populations and Alrasasimah & Alsabaani [15] in the Saudi context, suggesting that income–self-care associations of a similar direction have been observed across diverse healthcare settings, although the strength and underlying mechanisms of these associations may differ by context.

Within the specific context of southern Riyadh’s primary healthcare system, this income gradient has direct implications for care delivery. Patients presenting at PHCs in this district are disproportionately concentrated in lower-income brackets, suggesting that the prevailing self-care environment is characterized by material constraints that limit engagement with recommended management practices. Nursing practitioners working in these settings must therefore adopt economically sensitive approaches to diabetes education and self-care support, for example, recommending low-cost dietary modifications that align with culturally familiar foods, facilitating access to subsidized monitoring supplies, and connecting patients with available social welfare resources [30,31,32]. At the systemic level, findings support the case for targeted investment in community-based diabetes support programs in economically disadvantaged districts [33,34,35].

### 4.2. Age-Related Vulnerability and Self-Care Disparities

Age showed the strongest sociodemographic correlation with diabetes self-care management in this study (rs = −0.49, 95% CI [−0.63, −0.31], *p* < 0.01), remaining independently significant after adjustment for income, education, and gender (Section 3.5), a finding of potential clinical and policy relevance given the expected demographic aging of the Saudi population, although the cross-sectional design precludes any causal or predictive interpretation. The pronounced decline in both self-care and health literacy across age groups, from scores of 4.81 and 37.2 in the youngest group to 2.21 and 27.1 in participants over 65 years, points toward a substantial and compounding vulnerability in older adults with diabetes that warrants dedicated programmatic attention. Multiple mechanisms may underlie this association, including cohort effects in formal education (with older adults less likely to have completed secondary schooling), age-related cognitive changes affecting information processing, higher rates of comorbidity limiting physical self-care capacity, and potentially reduced access to digital health information resources among older generations.

From a nursing theory perspective, this pattern can be understood within Orem’s Self-Care Deficit Nursing Theory as a progressive erosion of self-care agency with advancing age, which may reflect age-related differences in health literacy and other factors not directly measured in this study [25]. The nursing response must correspondingly shift from an educational to a supportive and compensatory orientation for older patients, emphasizing simplified treatment regimens, frequent nurse-initiated follow-up, family and caregiver engagement, and use of visual and auditory (rather than text-heavy) educational materials [36,37,38]. The development and evaluation of age-specific diabetes self-care support programs within Saudi Arabian PHCs represents an important direction for future nursing research and practice innovation.

### 4.3. Educational Attainment: A Bivariate Association That Attenuates After Adjustment

Educational attainment showed significant, weak-to-moderate positive bivariate associations with both self-care management and health literacy in this study. However, as reported in Section 3.5, both associations attenuated substantially in the multivariable model once income and age were accounted for, becoming only marginal for self-care management (*p* = 0.056) and clearly non-significant for health literacy (*p* = 0.707). This pattern indicates that education’s bivariate association with these outcomes in this sample overlaps considerably with income and age, rather than reflecting a robust independent pathway. The following mechanisms are proposed as hypotheses that could explain the original bivariate pattern; because mediating variables such as social networks and healthcare-system familiarity were not directly measured or tested in this study, they should not be interpreted as demonstrated pathways, and the adjusted results should be treated as the more reliable indicator of education’s associations in this sample. Possible mechanisms through which education’s unadjusted association with self-care and health literacy could partly reflect its overlap with income and age include: higher education being associated with higher-paying employment; younger cohorts in this sample having both more formal schooling and more recent, less complicated diabetes self-management training; and greater financial resources enabling access to self-care materials and healthcare services independently of formal health literacy. Future research with a larger sample could examine whether an education × income or education × age interaction better characterizes this relationship than the additive model used here.

### 4.4. Gender and Self-Care and Health Literacy Outcomes

Gender was not significantly correlated with either diabetes self-care management (rs = 0.11, 95% CI [−0.10, 0.31], *p* > 0.05) or health literacy (rs = −0.02, 95% CI [−0.22, 0.19], *p* > 0.05) in this sample, and remained non-significant after adjustment for income, age, and education in the multivariable model (Section 3.5). This null finding contrasts with some international literature reporting gender differences in diabetes self-management, and it may reflect features specific to the Saudi and broader Gulf family context. In many Saudi households, diabetes self-care activities such as meal preparation, medication reminders, and clinic scheduling are frequently shared or family-mediated rather than managed individually, which could attenuate gender-based differences that are more pronounced in settings where self-care is an individual undertaking. It is also possible that the relatively homogeneous socioeconomic profile of participants recruited from southern Riyadh PHCs limited the variability needed to detect a gender effect. These explanations are offered as hypotheses for future research rather than as findings supported by the present data, since family-structure and household-role variables were not directly measured in this study. Whether these patterns differ in other Saudi regions with different educational or age profiles is an open question that the present cross-sectional, single-region design cannot address.

### 4.5. Implications for Equity-Focused Nursing Practice

The collective pattern of findings in this study, whereby income, age, and education emerge as significant sociodemographic stratifiers of diabetes self-care, with income inequity and age-related vulnerability as the most pronounced, calls for a fundamentally equity-oriented approach to diabetes nursing in southern Riyadh PHCs. Equity-focused nursing practice requires that care providers move beyond a uniform, information-transmission model of diabetes education toward a model that actively identifies and responds to the social circumstances shaping each patient’s self-care capacity [3]. This includes conducting socially informed needs assessments that elicit patients’ material circumstances, social support resources, and literacy levels as part of routine diabetes care; tailoring educational interventions to patients’ specific socioeconomic contexts; advocating for policy reforms that address the structural income and educational inequities constraining self-care; and partnering with community organizations to deliver supplementary self-care support in low-income neighborhoods.

### 4.6. Limitations and Future Directions

This study has several limitations. Its cross-sectional design precludes causal inference, and purposive sampling from PHCs in southern Riyadh limits the generalizability of the findings to other Saudi populations. Although multivariable analyses confirmed that income and age remained independently associated with both self-care management and health literacy, education became marginal or non-significant and gender remained non-significant after adjustment. The models explained a moderate proportion of variance (adjusted R^2^ = 0.34 for self-care management and 0.22 for health literacy), indicating that other unmeasured factors also contribute to these outcomes.

Self-reported self-care data may be affected by social desirability and common-method bias. Important factors, including comorbidities, social support, psychological distress, healthcare utilization, and objective clinical indicators such as HbA1c, were not assessed. The examination of multiple bivariate correlations without adjustment may also increase the risk of Type I error. In addition, excluding participants with missing data, the small > 65 years subgroup, and the exclusion of individuals with psychiatric diagnoses may have introduced selection bias and further limited representativeness. Although non-normal residuals in the health-literacy model were addressed using bootstrap confidence intervals, its estimates should still be interpreted cautiously. Future longitudinal and geographically diverse studies should include broader clinical and psychosocial factors and evaluate targeted, socially responsive self-care interventions.

## 5. Conclusions

This study contributes to our understanding of the social patterning of diabetes self-care management and health literacy among adults in southern Riyadh by examining both the bivariate associations and, through multivariable regression, the adjusted associations of income, age, educational attainment, and gender with these outcomes through a WHO CSDH framework. Income and age were independently associated with both self-care management and health literacy after adjustment for the other three variables; educational attainment showed significant bivariate associations with both outcomes, but these attenuated to marginal or non-significant after adjustment, suggesting they are not independent of income and age. Gender was not significantly associated with either outcome at either the bivariate or adjusted level. Because the design is cross-sectional, these associations should not be interpreted as evidence of causal effects, and the moderate proportion of variance explained by the adjusted models (22–34%) indicates that other, unmeasured factors also contribute substantially to both outcomes. The observed patterns support equity-focused, structurally informed nursing interventions that prioritize income and age as the sociodemographic factors most robustly associated with self-care and health literacy disparities in Saudi Arabian urban–peripheral communities.

## Figures and Tables

**Table 1:** Spearman Correlations Between Sociodemographic Variables, Self-Care Management, and Health Literacy (*n* = 92).

Variable	Self-Care Management (rs)	Health Literacy (rs)	Direction
Income Level	0.40 ** [0.21, 0.56] (<0.01)	0.41 ** [0.22, 0.57] (<0.01)	Positive
Age	−0.49 ** [−0.63, −0.31] (<0.01)	−0.36 ** [−0.53, −0.17] (<0.01)	Negative
Education Level	0.29 ** [0.09, 0.47] (<0.01)	0.26 * [0.06, 0.44] (<0.05)	Positive
Gender	0.11 [−0.10, 0.31] (>0.05)	−0.02 [−0.22, 0.19] (>0.05)	Non-significant

rs = Spearman rank-order correlation coefficient; bracketed values are 95% confidence intervals for rs (Fisher z). ** *p* ≤ 0.01; * *p* ≤ 0.05.

**Table 2:** Diabetes Self-Care Management and Health Literacy by Income Group (*n* = 92).

Income Group	Self-Care M	Health Literacy M
<5000 SAR (Low)	2.84	29.4
5000–9999 SAR (Middle)	3.71	33.2
≥10,000 SAR (Higher)	4.93	38.1

SAR = Saudi Arabian Riyals. Self-care scores represent mean frequency of self-care activities across a 7-day recall period (range: 0–7). Health literacy scores derived from HLS-Q12 (range: 12–48).

**Table 3:** Diabetes Self-Care Management and Health Literacy by Age Group (*n* = 92).

Age Group	*n* (%)	Self-Care M (SD)	HL M (SD)
18–35 years	26 (28.3%)	4.81 (1.24)	37.2 (5.1)
36–50 years	33 (35.9%)	3.62 (1.31)	33.7 (5.8)
51–65 years	22 (23.9%)	2.94 (1.12)	30.4 (6.2)
>65 years	11 (12.0%)	2.21 (0.98)	27.1 (5.9)

HL = Health Literacy. SD = Standard Deviation. Self-care scores represent mean frequency of self-care activities (range: 0–7). Health literacy scores derived from HLS-Q12 (range: 12–48).

**Table 4:** Multivariable Linear Regression Predicting Self-Care Management and Health Literacy from Sociodemographic Variables (*n* = 92).

Predictor	B (SDSCA)	β (SDSCA)	*p* (SDSCA)	B (HLSQ12)	β (HLSQ12)	*p* (HLSQ12)
Income (SAR/month)	8.5 × 10^−5^	0.295	0.002	4.0 × 10^−4^	0.350	0.001
Age (years)	−0.045	−0.401	<0.001	−0.138	−0.306	0.002
Education level	0.261	0.175	0.056	0.223	0.037	0.707
Gender (female = 1)	0.348	0.107	0.221	−0.602	−0.046	0.629

B = unstandardized coefficient; β = standardized coefficient; SDSCA = self-care management; HLSQ12 = health literacy. Self-care model: R^2^ = 0.367, adjusted R^2^ = 0.338, F(4,87) = 12.60, *p* < 0.001. Health literacy model: R^2^ = 0.256, adjusted R^2^ = 0.222, F(4,87) = 7.49, *p* < 0.001. All variance inflation factors < 1.2.

## Data Availability

The results presented in this study are contained within the article. De-identified individual participant data are available from the corresponding author upon reasonable request, subject to institutional and ethical approval requirements.
